# Acceptability of a Web-Based Financial Education Intervention for Latino Caregivers: Mixed Methods Evaluation

**DOI:** 10.2196/70471

**Published:** 2025-07-15

**Authors:** Susanna Mage, Donna Benton, Kathleen Wilber, Rocio Aguila Rodriguez, David Silva, Alexander Gonzalez, Frank Puga, Kylie Meyer

**Affiliations:** 1 Leonard Davis School of Gerontology, University of Southern California, 3715 McClintock Avenue, Los Angeles, CA, 90089, United States, 1 213 740 5156; 2 Frances Payne Bolton School of Nursing, Case Western Reserve University, Cleveland, OH, United States; 3 School of Nursing, University of Alabama at Birmingham, Birmingham, AL, United States

**Keywords:** informal caregiving, caregiver stress, dementia, disparities, financial strain, ethnicity, acceptability, intervention, Latino, Latina

## Abstract

**Background:**

Latino caregivers of persons living with dementia face distinct financial challenges, spending nearly half of their annual household income on caregiving expenses, almost twice as much as non-Latino caregivers. Cultural norms, limited access to financial resources, and underrepresentation in intervention research compound this economic burden. There remains a critical gap in evidence-based, culturally tailored programs designed to reduce financial stress among Latino caregivers.

**Objective:**

This study examines the acceptability and perceived value of the Confidently Navigating Financial Decisions and Enhancing Financial Well-Being in Dementia Caregiving (CONFIDENCE) program, a culturally tailored, web-based psychoeducational intervention designed to reduce financial stress and improve financial well-being among Latino caregivers of persons living with dementia.

**Methods:**

We applied a multimethod approach to evaluate the acceptability of the CONFIDENCE pilot. Following completion, 14 caregivers participated in in-depth, semistructured interviews, and 27 (39% of program participants) completed a 14-item satisfaction survey. The study was guided by a theoretical framework of acceptability composed of 7 domains: affective attitude, burden, ethicality, intervention coherence, opportunity costs, perceived effectiveness, and self-efficacy. Interview transcripts were analyzed independently by 4 coders (SM, RAR, DS, and KM) using thematic analysis, and survey responses were summarized using descriptive statistics.

**Results:**

Participants overall reported satisfaction with the CONFIDENCE program. Qualitative analysis of interview transcripts revealed four themes: (1) the perceived need for financial intervention; (2) perceived intervention effectiveness, particularly in improving financial knowledge, communication skills, and mental health; (3) positive emotional responses to participation, with high praise for the program’s group setting and cultural relevance; and (4) recommendations for intervention improvement, including shorter sessions and technology support. Survey results confirmed high acceptability, with 96% (n=26) of respondents reporting they enjoyed the program. All participants (n=27, 100%) said they would recommend CONFIDENCE to other Latino caregivers. Participants reported improved awareness of available resources, increased confidence in financial decision-making, and reduced financial stress. Caregivers appreciated the group setting, which allowed for mutual interaction and learning; discussions fostered emotional connection and peer learning. Participants praised the content’s trustworthiness and relevance. Although most agreed that participation required minimal effort, barriers such as time constraints and technology challenges were noted.

**Conclusions:**

Findings suggest that CONFIDENCE is acceptable among Latino caregivers of persons living with dementia. Culturally tailored content, group learning, and an emphasis on practical financial strategies were central to the program’s success. Findings will inform program refinements, including limiting burden by addressing barriers while maintaining strengths such as group learning to optimize engagement. Considering the unique economic burdens Latino caregivers face, it is vital to develop and support interventions tailored to their unique needs. This study adds to the limited but growing literature on financial well-being interventions for Latino caregivers and highlights the value of culturally informed, community-driven approaches to supporting caregiver financial resilience.

## Introduction

### Background

Family caregivers play a crucial role in supporting older adults living with chronic illness and disability; yet, many face significant economic challenges. Caregivers of persons living with dementia experience substantial out-of-pocket expenses due to medical copayments, necessary home modifications, specialized caregiving supplies, personal care services, and home care. These costs precipitate far-reaching economic and psychological consequences, including financial strain and anxiety [1-3]. Households of individuals diagnosed with dementia have been shown to experience more than a 2-fold increase in out-of-pocket health care spending and a decline in net worth exceeding 60% within 8 years of diagnosis [2]. This kind of financial insecurity can have both immediate and long-term effects on caregivers’ ability to meet basic needs, career development, generational wealth, and overall economic well-being [4,5].

The financial burden of caregiving is acutely evident among resource-limited populations disproportionately impacted by dementia [6], with recent findings showing that out-of-pocket costs and related financial strain disproportionately affect historically marginalized groups, including racial and ethnic minority populations [4]. Latino caregivers are particularly vulnerable to these financial pressures [7,8]. This is due in part to delayed access to services, more unpaid care responsibilities, and limited availability of culturally tailored support. Despite depending heavily on informal care and choosing more affordable health care options (ie, adult day care over nursing facilities) [8], Latino families of persons living with dementia still incur higher costs than non-Latino families [7,9]: on average, caregivers spend 26% of their annual household income on caregiving, whereas Latino caregivers spend nearly twice as much (47%) [3]. One study found that Mexican American caregivers contend with caregiving burdens that are intensified by socioeconomic constraints and disrupted life opportunities, contributing to greater financial strain and worse physical and mental health outcomes [8].

Understanding values and norms central to Latino culture is crucial for addressing needs and developing financial support programs. Cultural values that influence approaches to caregiving and spending patterns include *Famalismo* and *Marianismo* [7,10-12]. *Famalismo*—prioritizing family welfare over individual desires—can contribute to career compromises that reduce potential earnings when a caregiver provides direct care over formal services. *Marianismo*—the characterization of women as family-centered and selfless—may reduce household income by Latinas’ prioritizing unpaid care roles over paid labor force participation. Within Latino families, caregiving expectations often fall disproportionately on women, which can limit their participation in the paid workforce and contribute to unequal caregiving burdens [7,8]. Caregivers under strain are more likely to consider skilled nursing facilities for their loved ones [13]. However, despite providing more intensive care, Latino caregivers historically prefer providing care in the community, possibly due to familial obligations and concerns about the quality of nursing facilities [8,11,14].

Strengthening caregivers’ financial security is a national priority in the United States, reflected in the first-ever National Strategy on Caregiving [15]. Financial education and training interventions have been found to improve money management and the overall well-being of participants [16], and several studies have shown the positive impact of psychosocial interventions in easing caregiver burden and mitigating distress [7,16-18]. Interventions tailored to meet participant needs, preferences, and values increase satisfaction and engagement [8,19,20]. Despite increasing recognition of the unequal caregiving burden felt among Latino families, most evidence-based interventions have not been adapted for their cultural needs, and few address financial strain directly [21].

### This Study

The Confidently Navigating Financial Decisions and Enhancing Financial Well-Being in Dementia Caregiving (CONFIDENCE) intervention was developed to reduce financial stress and lower Latino caregivers’ out-of-pocket spending on care costs by displacing such spending with community and informal resources and facilitating help-seeking behaviors (personal communication by Dr Kylie Meyer, 2024). This study aims to test the acceptability of its pilot program using Sekhon’s theoretical framework of acceptability (TFA) as a guide [20]. Evaluating acceptability can identify factors affecting adherence, execution, and outcomes, facilitating necessary adjustments to the intervention’s design to boost its practicality and acceptance. The TFA breaks acceptability into 7 validated components: affective attitude, burden, perceived effectiveness, ethicality, intervention coherence, opportunity costs, and self-efficacy, defined further in Multimedia Appendix 1. We selected this framework to guide our study because it provides a comprehensive structure for assessing emotional, cognitive, and practical dimensions of intervention acceptability. Given the strong influence of cultural values, caregiving expectations, and financial vulnerabilities within Latino communities, it was important to use a framework that could not only capture general satisfaction with the program but also be in alignment with participants’ values and perceived burdens. The TFA’s multidimensional focus makes it well-suited to evaluate culturally tailored interventions like CONFIDENCE.

## Methods

### Study Design

This study used qualitative and quantitative methods to examine the perspectives of Latino communities caring for persons living with dementia who participated in CONFIDENCE. Data were collected through in-depth, semistructured qualitative interviews (n=14) and a 14-item quantitative satisfaction survey (n=27). The TFA guided the study design, including the interview guide, coding, and interpretation processes [20].

### Intervention

#### Overview

The CONFIDENCE intervention is a culturally tailored 5-session psychoeducational program delivered via teleconference to up to 12 caregivers at a time. Sessions include the delivery of didactic content interspersed with practice exercises and discussions. The program was facilitated by interventionists who self-identified as Latino and, when delivered in Spanish, were native Spanish speakers. After each session, caregivers were encouraged to practice what they learned. Topics included navigating and seeking help from formal and informal resources, balancing employment and caregiving, and basic money management skills applied to caregiving (eg, using a budget to make financial decisions related to caregiving). To ensure that content was culturally tailored, the investigator team drew upon findings from qualitative interviews with Latino family caregivers about their experiences managing caregiving-related financial strain [22] alongside ongoing guidance from a community advisory council. The council provided input on key elements, including the length of discussion sessions and culturally appropriate phrasing to describe practice activities. A more detailed description of the cultural tailoring process can be found in Meyer et al [23]. The CONFIDENCE intervention was delivered at a community-based organization 11 times, including twice in Spanish, from 2022 to 2023.

#### Steps to Support Intervention Fidelity

Intervention fidelity was supported through the use of a structured facilitator guide, standardized slide decks to guide group discussion sessions, and template reminder emails encouraging participants to complete recommended between-session activities. All facilitators had at least a bachelor’s degree and prior experience providing support services to family caregivers within the service setting where the intervention was delivered. The lead facilitator, who helped develop the intervention and had prior personal caregiving experience, led the training for other facilitators. This training included role-playing of the intervention delivery, supervised delivery of at least 2 sessions prior to independently delivering sessions, and fidelity procedures. To monitor fidelity, an independent observer used a standardized checklist to review recordings from 5 randomly selected sessions. All observed sessions were delivered in the intended sequence, with at least 80% of intervention steps completed according to the facilitator guide. Facilitators also submitted attendance reports to the evaluation team, which were reviewed to help monitor participant engagement. Weekly evaluation team meetings, including the lead facilitator, and ongoing review of attendance and fidelity reports by the principal investigator (KM) helped address delivery issues, such as low participation during sessions.

### Data Collection

#### Eligibility and Recruitment

The CONFIDENCE program was delivered as a part of routine services at a community-based organization that regularly provides caregiver education and support. Approximately 60% of caregiving clients at this organization identify as Latino, and nearly 40% care for persons living with dementia. Caregivers were primarily informed about CONFIDENCE through email “blasts” and staff referrals. In total, 20 caregivers participated in an optional pre- and posttest study, which required registration through the organization’s web-based event platform. To support study recruitment, the program was also promoted more broadly, including flyers and social media posts. For more information on the recruitment methods used in the feasibility study, see Meyer et al [23].

A satisfaction survey with 14 items was sent by email to caregivers who registered for CONFIDENCE on the web-based event platform (ie, convenience sample) and met the following eligibility criteria: self-identified as Latino, provided care to persons living with dementia, 18 years or older, and attended at least 1 of the 5 CONFIDENCE sessions. Those who completed the satisfaction survey were also asked if they would be willing to complete a qualitative interview. A purposeful sample of 14 caregivers was recruited via email for qualitative interviews, including those who completed the satisfaction survey and indicated that they would be willing to be contacted, as well as feasibility study participants. Participants were selected to include caregivers of various genders, ages, geographic locations, and kin relationships with the care recipient.

#### Interview Guide

The TFA and recent literature on Latino caregivers informed the qualitative interview’s open-ended questions [20,22]. The research team reviewed the interview guide (Multimedia Appendix 2), which discussed the program’s purpose, the skills caregivers believed they acquired, how participants perceived it helped protect financial well-being, which parts were most helpful, and barriers and challenges to participation.

#### Interviews

Participants provided verbal consent to participate in individual interviews, which were conducted in Spanish or English, based on the participant’s language preference, via Zoom (Zoom Video Communications; 2022‐2023). All interviews were recorded and then transcribed by a professional transcription service, which also translated Spanish transcriptions. The interview method followed the recommendations of Baker and Edwards [24] to include at least 12 interviews until no new themes or codes emerged (ie, thematic saturation) [25], which was achieved after the 10th interview.

#### Satisfaction Survey

A 14-item satisfaction survey was sent to participants after the last session of the program (Table 1). The survey assessed the acceptability, overall perception of the program, and participants’ evaluation of the information presented. Satisfaction levels were rated using a 5-point Likert scale (1=strongly disagree, 2=disagree, 3=neutral, 4=agree, and 5=strongly agree). For each item, responses were categorized as “satisfied” if the participant selected “agree” or “strongly agree.” This survey was a newly developed tool grounded in Sekhon’s TFA [20].

**Table 1.** Survey items used to assess the acceptability of the CONFIDENCE^a^ intervention, aligned with seven domains of Sekhon’s theoretical framework of acceptability.

Construct of the theoretical framework of acceptability	Questions asked
Affective attitude: how an individual feels about the intervention	I felt comfortable and included in the CONFIDENCE program. I felt comfortable sharing about my caregiving experience and personal finances during the CONFIDENCE program. I enjoyed participating in the CONFIDENCE program. I trusted the information I received in the CONFIDENCE program.
Burden: the perceived amount of effort that is required to participate in the intervention	It was easy to understand the information presented during the CONFIDENCE program. The pace of the program was just right (ie, not too fast and not too slow). I found it easy to access the CONFIDENCE program on the web, including using Zoom.
Ethicality: the extent to which the intervention has a good fit with the individual’s value system.	The information presented in the CONFIDENCE program applied to my caregiving situation.
Intervention coherence: the extent to which the participant understands the intervention and how it works	I knew who to contact if I had any questions about the CONFIDENCE program. Overall, I found the program to be interactive.
Opportunity costs: the extent to which benefits, profits, or values must be given up to engage in the intervention	Overall, I found the program to be convenient to attend.
Perceived effectiveness: the extent to which the intervention is perceived as likely to achieve its purpose	I learned from the other caregivers whom I attended the program with. I would recommend for specifically other Latino or Latinx or Hispanic caregivers to participate in the CONFIDENCE program.
Self-efficacy: the participant’s confidence that he or she can perform the behaviors required to participate in the intervention	I found that take-home assignments added to my learning.

a CONFIDENCE: Confidently Navigating Financial Decisions and Enhancing Financial Well-Being in Dementia Caregiving.

### Data Analysis

We applied thematic analysis to examine interview transcripts [26]. Codes were devised to address each component of acceptability from Sekhon’s TFA, facilitating triangulation between qualitative findings and survey responses (Table 2). Four research team members (SM, RAR, DS, and KM) independently coded transcripts using NVivo (version 1.7.1; Lumivero). After initial coding, the team met with the lead coder (SM) for peer debriefing sessions. Discrepancies identified were resolved through discussion after each round, leading to iterative refinements of the codebook. As new codes were identified, like those related to culture, they were incorporated into the codebook and retrospectively applied to earlier transcripts. New codes and unresolved discrepancies were discussed with KM, who was available to adjudicate coding disagreements; however, no such adjudication was ultimately required. To assess participant satisfaction, we summarized survey responses using means and percentages.

**Table 2.** Codebook used for thematic analysis of qualitative interviews with Latino caregivers of persons living with dementia, grounded primarily in Sekhon’s theoretical framework of acceptability [20], with a supplemental inductive code to capture culturally specific relevance.

Codebook	Definition
Affective attitude	Caregiver expresses how they feel about the intervention.
Burden	Caregiver describes the perceived amount of effort that was required to participate in the intervention.
Ethicality	Caregiver indicates that the intervention is a good fit with their value system.
Intervention coherence	Caregiver shows an understanding of the intervention and how it works.
Opportunity costs	Caregiver reports that they had to give up benefits, profits, or values to engage in the intervention.
Perceived effectiveness	Caregiver perceives the intervention as likely to achieve its purpose.
Self-efficacy	Caregiver’s confidence that they can perform the behaviors required to participate in the intervention.
Relevance of Latino cultural and traditional values	Caregiver describes caregiving in relation to a sense of duty to their family (ie, Familialism) or traditional gender roles.

### Ethical Considerations

The University of Southern California institutional review board (IRB) reviewed the study protocol and issued an exempt determination (UP-23‐00020) for the secondary analysis of deidentified data. Data collected from the primary study were approved under 2 separate protocols by the Case Western Reserve University IRB: an expedited protocol (STUDY20221010) for caregivers enrolled in the optional pre- and posttest study and an exempt protocol (STUDY20221415) for caregivers completing the CONFIDENCE program outside the pre- and posttest study. Caregivers who were eligible for the pre- and posttest study provided written consent to participate prior to enrollment. For participants not enrolled in the study who were invited to complete the satisfaction survey, consent was implied upon survey completion following a review of an information sheet. Caregivers who participated in qualitative interviews gave verbal consent after reviewing a study information sheet. All consent forms and information sheets stated that deidentified data may be retained for future use. The University of Southern California IRB did not require additional consent for the use of deidentified data shared from Case Western Reserve University. Participants in the optional pre- and posttest study received up to $25 USD in Amazon gift card value, which included compensation for completing the satisfaction survey. Caregivers who participated in a qualitative interview received a $25 USD gift card, and those not enrolled in the study but who completed a satisfaction survey were given the option to provide their email address to receive a $25 USD gift card.

## Results

### Sample Characteristics

In total, 14 Latino caregivers of persons living with dementia participating in CONFIDENCE between May 2022 and September 2023 consented to an interview following completion of the program (Table 3); 11 (79%) in English and 3 (21%) in Spanish. The mean age of participants was 60 (SD 7) years. Most were women (95%). Nearly two-thirds (64%) had attended at least 3 CONFIDENCE sessions; roughly half (44%) completed all sessions.

**Table 3.** Demographic characteristics of Latino caregivers of persons living with dementia (n=14) who participated in qualitative interviews following the CONFIDENCE^a^ intervention.

Age (years)	Gender	Below 250% FPL^b^	Language	Sessions attended
52	Woman	Yes	English	4
66	Woman	No	English	1
62	Woman	No	English	5
56	Woman	Yes	English	4
56	Woman	No	English	5
54	Woman	No	English	4
57	Woman	Yes	Spanish	5
46	Woman	—^c^	Spanish	5
63	Woman	—	English	2
73	Man	No	English	3
59	Woman	Yes	Spanish	2
63	Woman	—	English	4
63	Woman	—	English	4
68	Woman	Yes	English	5

a CONFIDENCE: Confidently Navigating Financial Decisions and Enhancing Financial Well-Being in Dementia Caregiving.

b FPL: federal poverty level.

c Missing data.

### Thematic Insights

Interview findings revealed four key themes: (1) perceived need for financial intervention, (2) perceived effectiveness of the intervention, (3) positive responses to participation, and (4) recommendations to improve the intervention.

#### Theme 1: Perceived Need for Financial Intervention

The first theme characterized the perceived need for a program like CONFIDENCE to address financial well-being and lower out-of-pocket care costs. Applying the TFA, this theme primarily reflects “intervention coherence” [20].

##### Caregivers Found the Content Applicable

Caregivers appreciated building financial management skills through CONFIDENCE, recognizing its importance: “It’s to help caregivers, especially those of us who have not managed our finances on our own, teach us and guide us on how to begin the process of taking over some of the skills of managing our finances” (Participant 3). They expressed eagerness for such a program. “It’s the class I was hoping for a long time,” shared one participant, “it was my New Year’s resolution to be more financially confident” (Participant 4). The unpredictable progression of dementia highlighted the need to save for future care costs:

Dementia can go on for many years. It doesn’t matter your financial situation. People have no clue how long it runs, what home care costs, or the financial issues if you need placement.

[Participant 14]

##### CONFIDENCE Is Relevant to the Latino Community

During interviews, caregivers acknowledged the program’s benefit for Latino families due to a perceived gap in financial knowledge, partially related to the division of household tasks between genders. They emphasized its value to women, who are often expected to be caregivers:

It is on a very personal level for us Mexican-Americans. Typically, the man takes over the finances ... so this program is very important ... whether it’s the wife or daughter, so she can learn how to handle finances.

[Participant 3]

One caregiver felt the program could “help primarily Hispanic families to deal with strategies for why our out-of-pocket expenses are so much higher than other groups” (Participant 11). Caregivers valued the program’s approach, which respects multiple ways of providing care rather than questioning care choices.

There’s a reason why we pay more out-of-pocket than other people. It’s a different mindset, and [CONFIDENCE] doesn’t say, “Stop doing that.” “Why are you doing what you’re doing?” Instead, it gives strategies and ideas.

[Participant 11]

### Theme 2: Perceived Effectiveness of the Intervention

Theme 2 reflected the TFA’s categories of “perceived effectiveness” and “self-efficacy” [20]. Analyses of interview transcripts indicated that participants believed they acquired new knowledge and skills to help them manage their financial situation.

#### Caregivers Felt CONFIDENCE Improved Their Knowledge of Finances and Resources

Participants, especially those new to managing household finances, shared increased awareness of spending and praised CONFIDENCE for making financial well-being information accessible: “This information is amazing. It’s here for us to dive in and have the confidence to explore it. This class did that; it gave me the confidence” (Participant 3). The program empowered caregivers to think of innovative solutions to financial challenges, as they became more aware of the wide range of available resources: “I was very surprised finding out how many resources are available” (Participant 6).

We didn’t know that [resources] were available, and, unfortunately, we didn’t know this was part of the stress that all caregivers experience when the sick person depends on you.

[Participant 8]

Several caregivers provided examples of successfully connecting with resources they learned about, including In-Home Supportive Services (Participant 5), adult daycare centers (Participant 1), and Medicaid workshops (Participant 12). Knowledge of community resources helped some caregivers offset the costs of caregiving:

I got so much help with providing [equipment] for my mom. I got a chair for the bath and grab-hold bars. I wasn’t aware that I qualified for $250 to buy stuff for my mom.

[Participant 5]

#### Caregivers Felt the Program Improved Their Financial Well-Being

Strategies covered in the program to help manage care-related costs included tracking expenses, using a budget to make care-related decisions, asking for help from external resources, and setting financial goals.

It gave different strategies that I can modify for what I need. Everybody is gonna be different, but the great thing that it gave was strategies and tips, and you use what you can. I came from a pretty high place, but this put me over the top.

[Participant 11]

Caregivers noted that the skills gained improved their financial habits: “It has helped me a lot to manage my expenses more reasonably” (Participant 7). Several caregivers credited goal-setting activities for improving finances; clear, achievable targets fostered savings and debt reduction:

I got my tax return. Paid off a credit card. Paid down two credit cards. Put money in my emergency thing. I knocked off three goals ... It was awesome to break down goals in a way where I knew for sure what I was gonna do and how it was gonna make me feel as a result.

[Participant 5]

#### Learning Communication Skills Helped Caregivers Get Their Needs Met

Learning communication skills and engagement strategies, such as how to seek help from service providers, helped caregivers access support to offset care-related expenses.

This class gave me the ability to retool how I speak to someone who would be able to help me with different resources to help my mom and me and language better to outside resources to help us as I move forward with her.

[Participant 4]

CONFIDENCE taught caregivers techniques for effectively discussing support needs with their social circle. Many successfully sought family assistance after learning to ask for help through the program: “It helped me to plan that I had to get help from my family ... And so, I did speak to my family, and now I got a little bit more help from them” (Participant 6). Skills related to help-seeking are particularly notable, given the perceived cultural reticence around money talk within Latino families, a “stigma,” as one caregiver described (Participant 4).

#### Caregivers Felt the Program Improved Mental Health

Interviews showed that participation in CONFIDENCE was believed to alleviate financial stress and improve mental and emotional well-being.

I was sobbing so much. Then, once I started applying, doing the little exercises, and reading the material, something shifted in me. Something had gone to sleep for a while ... And what it did was it–it recharged it. It woke that problem-solving part of my brain again.

[Participant 5]

CONFIDENCE reinforced self-confidence and provided assurance: “It reassured me, yeah, you are going in the right direction” (Participant 10). Another caregiver echoed, “It helped reassure me, hey, you have these skills. You can do that” (Participant 12). Caregivers felt validated in their decision-making and equipped to handle surprises and assert their needs.

### Theme 3: Positive Responses to Participation

In alignment with the “ethicality” and “affective attitude” TFA domains [20], caregivers expressed a favorable opinion toward the intervention, indicating that it aligned well with their values and could benefit other caregivers.

#### Caregivers Enjoyed Participating in CONFIDENCE

Caregivers expressed satisfaction with the program and that they would recommend other caregivers to enroll: “This class is incredible. It’s an amazing gift because of this class” (Participant 4). “It was very successful for those of us in the class,” said one caregiver. “I think it would be great for any caretaker” (Participant 1). Others conveyed a desire to re-enroll and to continue with training.

Honestly, there’s nothing negative to say. I’d like to take the class again after a year ‘cause there’s always new things going on. I try to take classes more than once because I always learn something.

[Participant 10]

#### Caregivers Valued the Group Discussions and Appreciated the Flexibility of the Program Design

Most caregivers felt solidarity with other participants and that this collective experience added to the program’s perceived impact. Participants reported learning from hearing about others’ financial problem-solving strategies.

Somebody shared a skill that was really good. Every time they get a $10 bill, they put it away for a whole year ... I started doing something like that with larger denominations, and I’ve saved a few hundred dollars.

[Participant 10]

Group sessions helped caregivers recognize similar challenges faced by others; they were not alone: “I was feeling stressed and sad because [I thought] I was the only one, but hearing other stories and learning that there are resources to help, besides family, it helped me emotionally” (Participant 6). This awareness fostered emotional support within the group, making participants more receptive to sharing and accepting financial guidance.

The program’s flexibility, which allows caregivers to access materials at their convenience both now and in the future, was well-received: “My mother’s dementia goes in stages. I don’t need this right now, but I am going to need it, and now I have a place to look when that moment comes” (Participant 7).

### Theme 4: Recommendations to Improve the Intervention

Caregivers were asked to reflect on their experiences and provide feedback, offering insights into potential modifications and improvements to the intervention. This feedback elucidated potential “burden” and “opportunity costs” [20] associated with participation.

#### Help Caregivers Manage the Demands on Time

Caregivers reported difficulty attending weekly discussion sessions: “Sometimes it’s difficult to come to class because our loved ones need our care,” shared one caregiver. Another caregiver shared that the length of sessions posed a barrier.

Two hours is hard for me. I can’t get that kind of time away from Mom. It would be better, I think, like an hour and a half or even an hour, but an hour might not give you enough time to get that energy going, you know?

[Participant 11]

Considering their already busy schedules, some caregivers found that the time required for take-home assignments was burdensome. They felt that the inability to complete tasks outside the designated course time could deter caregivers from joining future sessions.

Sometimes people are just burnt out. That might be what causes people to fall out of coming, because they feel like, “I was supposed to do an assignment, and I didn’t do it. I’m embarrassed.”

[Participant 5]

#### Facilitate Engagement During Group Sessions

Although participants were enthusiastic about interacting with fellow caregivers, some felt that the facilitators could have been more engaging: “I had the experience of being more talked at than engaged. It was a lot of reading off the presentation ... You have to be engaged to be engaging” (Participant 2). Some participants observed a reluctance among peers to participate in discussions. “Trying to get most of the people engaged was rough,” said one caregiver, “but it’s always gonna be rough. Some people are gonna sit there and listen, and pick things up as opposed to getting involved” (Participant 9).

#### Address Technology-Related Issues

Caregivers with limited technological proficiency encountered challenges that hindered participation in the web-based program: “Since I haven’t learned to use a computer very well, I missed several sessions” (Participant 13). Other technological barriers affecting program delivery, like poor audio quality during video-sharing, also impeded caregivers’ engagement. “We want to get every piece that they’re saying and discussing. If the audio is poor, it loses it” (Participant 4).

### Satisfaction Survey

Following the intervention, 27 of 69 (39%) caregivers who participated in the CONFIDENCE program completed satisfaction surveys. Most were women (78%) and lived with the care recipient (70%), 74% were children or children-in-law, and 26% were spouses or partners. In total, 25 (93%) respondents identified that they had served as the primary caregiver for over 2 years. Near half (41%) provided 15 or more care hours per week; 48% were employed. Additional respondent characteristics can be found in Table 4.

**Table 4.** Demographic characteristics of Latino caregivers of persons living with dementia (n=27) who completed a satisfaction survey following participation in the CONFIDENCE^a^ program.

Gender	State	Maritalstatus	Education	Employment status	Relationship to CR^b^	Live with CR	Hours of care per week	Years of care
Woman	CA	Partner	Some college	Working (self-employed)	Parent or PIL^c^	No	4	2‐5
Man	AZ	Married	College or more	Retired	Spouse or partner	Yes	8	1‐2
Woman	CA	Never married	College or more	Working (paid employee)	Parent or PIL	Yes	6	5+
Woman	CA	Divorced	Some college	Not working—looking for work	Parent or PIL	Yes	24	2‐5
Man	CA	Married	College or more	Retired	Parent or PIL	No	13	1‐2
Woman	CA	Married	College or more	Working as a paid employee	Parent or PIL	No	2	5+
Man	TX	Never married	College or more	Not working—other	Parent or PIL	Yes	24	—^d^
Woman	TX	Married	College or more	Retired	Spouse or partner	Yes	18	5+
Woman	CA	Divorced	College or more	Working, self-employed	Parent or PIL	Yes	24	2‐5
Woman	CA	Married	Some college	Full-time student or trainee	Parent or PIL	No	12	5+
Woman	CA	Married	Some college	Working as a paid employee	Parent or PIL	Yes	6	2‐5
Woman	NJ	Never married	College or more	Not working—looking for work	Parent or PIL	Yes	1	2‐5
Woman	CA	Separated	Some college	Working, self-employed	Parent or PIL	Yes	17	5+
Woman	—	Never married	Some college	Working as a paid employee	Parent or PIL	No	12	2‐5
Man	CA	Married	HS^e^ or less	Retired	Spouse or partner	Yes	24	2‐5
Woman	CA	Widowed	Some college	Working as a paid employee	Parent or PIL	Yes	16	1‐2
Woman	—	Never married	Somecollege	Disabled	Parent or PIL	Yes	15	5+
Woman	CA	Married	College or more	Working as a paid employee	Parent or PIL	No	2	5+
Woman	CA	Married	College or more	Retired	Spouse or partner	Yes	16	5+
Man	CA	Married	Some college	Working as a paid employee	Parent or PIL	No	4	2‐5
Woman	—	Married	Some college	Retired	Spouse or partner	Yes	5	2‐5
Woman	CA	Never married	HS or less	Working as a paid employee	Parent or PIL	Yes	—	2‐5
Woman	AZ	Never married	Somecollege	Retired	Parent or PIL	Yes	12	5+
Woman	CA	Married	Somecollege	Retired	Spouse or partner	Yes	24	5+
Woman	AZ	Married	Somecollege	Retired	Parent or PIL	No	4	1‐2
Woman	—	Never married	HS or less	Working as a paid employee	Parent or PIL	Yes	—	2‐5
Man	CA	Married	Some college	—	Spouse or partner	Yes	24	2‐5

a CONFIDENCE: Confidently Navigating Financial Decisions and Enhancing Financial Well-Being in Dementia Caregiving.

b CR: care recipient.

c PIL: parent-in-law.

d Missing data.

e HS: high school.

**Figure 1.**
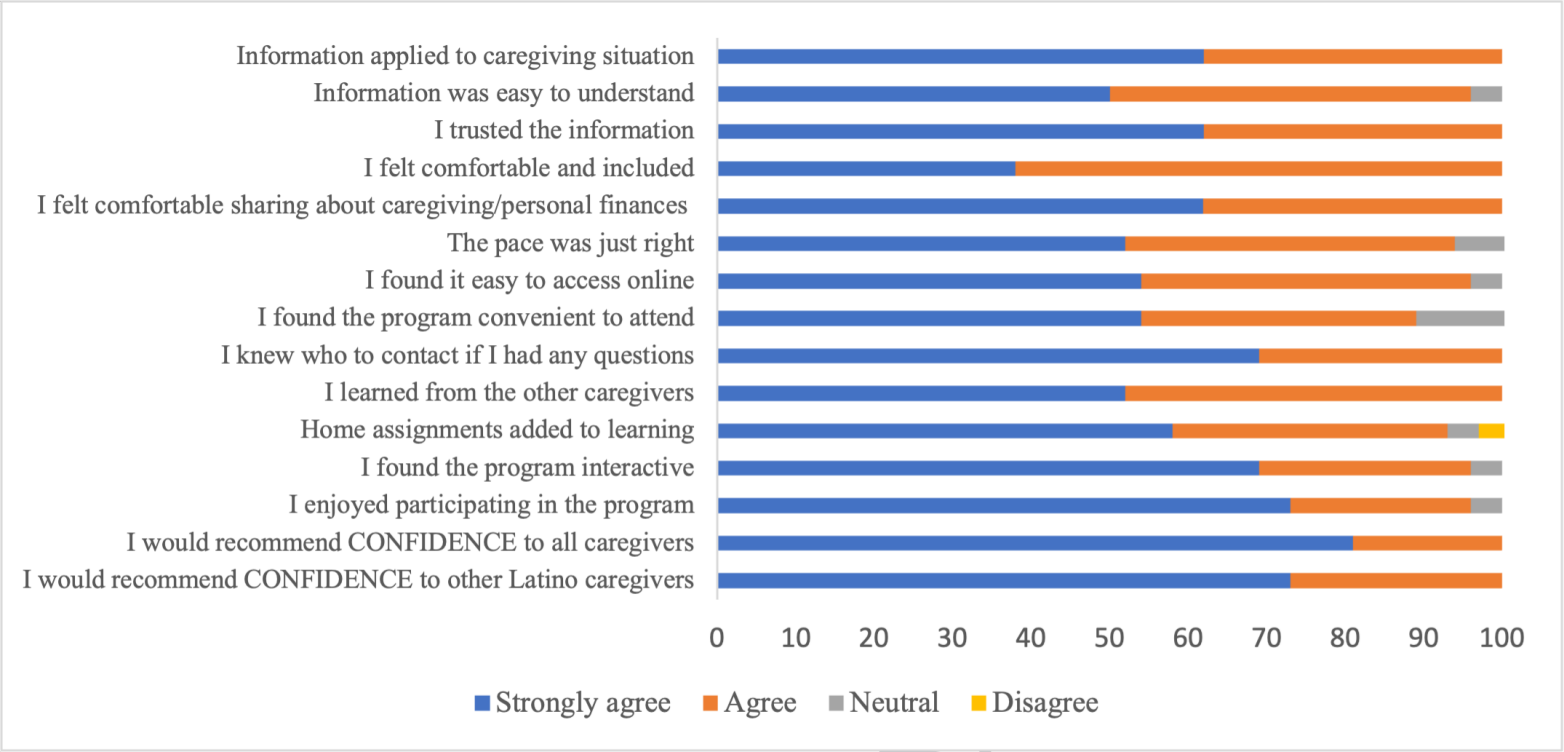
Ratings from 27 Latino caregivers of persons living with dementia on key program acceptability domains, based on a 14-item postintervention satisfaction survey following participation in the CONFIDENCE intervention. The “strongly disagree” response option was not selected by any participant and is therefore excluded. CONFIDENCE: Confidently Navigating Financial Decisions and Enhancing Financial Well-Being in Dementia Caregiving.

Caregivers expressed high satisfaction with CONFIDENCE’s content, setting, and delivery. All respondents (27/27, 100%) agreed (ie, responding “agreed” or “strongly agreed”) that they would recommend the program to all caregivers and specifically to Latino caregivers. All respondents (27/27, 100%) agreed that the information presented was trustworthy and applied to their caregiving situation and that they felt comfortable sharing personal experiences and financial details. Nearly all caregivers (26/27, 96%) enjoyed participating in the program and found the content easy to understand. Most respondents (25/27, 93%) found CONFIDENCE easy to access via Zoom, with nearly 90% (24/27, 89%) responding that it was convenient to attend the group discussion sessions. Additionally, most respondents (25/27, 93%) agreed that take-home assignments added to their learning. A full summary of the survey results is presented in Figure 1.

## Discussion

### Principal Findings

This study examined the acceptability of a financial psychoeducation program, CONFIDENCE, for Latino caregivers of persons living with dementia by assessing its perceived value, effectiveness, and participants’ overall satisfaction. Feedback was gathered on the program’s structure, relevance, most beneficial aspects, and suggestions for improvement. Caregivers endorsed the program, reporting increased financial awareness and high satisfaction with its content and delivery. Quantitative survey data corroborated qualitative findings, indicating that caregivers found the intervention engaging, understandable, relevant, and trustworthy (Figure 1). The use of an underlying framework (Sekhon’s TFA) facilitated the evaluation of the overlap between qualitative and quantitative findings. Survey responses and interviews reflected comfort and willingness to discuss caregiving experiences and financial situations. In addition, participants shed light on areas of the intervention that might need modification or improvement.

### Financial Interventions for Caregivers

Caregiver interventions seldom address financial well-being [27], despite evidence that most caregivers are unprepared for health care expenses not covered by insurance. The CONFIDENCE program targeted caregivers from the Latino community who generally experience even higher levels of financial strain than the average caregiver population [3]. Content for the CONFIDENCE financial psychoeducational intervention was developed based on interviews with Latino caregivers and iterative feedback from a community advisory board. These strategies followed best practices in culturally tailoring interventions and likely enhanced the program’s acceptability by ensuring that content was relevant to caregivers [28-30]. CONFIDENCE also incorporated proven strategies such as service systems and resource navigation, financial education, and asset-building to alleviate financial strain and enhance financial well-being [27].

### Acceptability of Psychoeducation Interventions for Caregivers

Despite different content and aims, caregiver satisfaction reports align with findings from the literature, highlighting the benefits of group-based programs for interactive problem-solving regarding preparing for the future, identifying and reorganizing the support team, and self-care [31]. Findings of high satisfaction suggest the perceived value of interactive problem-solving among caregivers, resonating with results from similar interventions for caregivers [31-33]. Communication skills training is considered essential for caregivers, as it helps strengthen emotional connections with other caregivers, family, and friends [34]. Including this type of training in CONFIDENCE fostered relationships and understanding between the caregivers during group sessions—and with their network of friends and family outside of the intervention to promote help-seeking behaviors. Active participation and skill-building increase financial and self-confidence among caregivers, supporting the TFA [20].

Previous studies have established the appeal of web-based interventions for family caregivers [32,33,35]. Although previously reported challenges of web-based interventions, like time constraints and scheduling conflicts, were also found in this study, participants were satisfied with the overall design. Like other digital group-based interventions, such as TeleSavvy, CONFIDENCE prioritized mental health, which helps create a safe, positive learning environment [36]. Mirroring the advantages of such programs, the hybrid model blended web-based group interactions with the flexibility of asynchronous delivery. Our research supports findings that peer support is beneficial for managing the complexities of financial well-being among caregivers, as it encourages open discussions on financial issues, enhancing the group’s collective problem-solving capacity [31,32]. CONFIDENCE’s flexible format, which allows caregivers to engage with materials at their own pace, was well-received and well-suited to varying schedules. The design facilitated independent learning, underscoring the effectiveness and practicality of self-paced learning models.

### Culturally Tailored Interventions for Latino Caregivers

Findings indicate that CONFIDENCE is an acceptable culturally tailored financial education program for Latino caregivers, a demographic with higher-than-average out-of-pocket care costs and a greater likelihood of limited formal financial engagement [3,37]. Latino caregivers may also face unique financial strains, often shouldering out-of-pocket expenses not covered by Medicare or Medicaid [7,11]. The US Latino population is both increasing and aging, adding to increases in dementia. Combined with lower use of formal care services and barriers to accessing care, overall expenses for Latino persons living with dementia and their caregivers are expected to increase at a faster rate than for non-Latino persons living with dementia [7,9]. There is a need for culturally targeted financial programs to address their higher levels of out-of-pocket costs [21,22,38].

Research acknowledges the importance of culturally informed support for caregivers belonging to communities where caregiving is heavily influenced by ethnic identity and cultural values [10,11,38,39]. However, many existing programs fail to meet the unique needs of diverse ethnic groups [21,40]. Preparing families to navigate available resources and financial decisions could mitigate financial risks while aligning with cultural norms about caregiving within the Latino community [7,11]. CONFIDENCE addresses the unique challenges of juggling caregiving with financial duties while acknowledging the preference for in-home care. Caregivers in this study reported that the program addressed the needs of Latino family caregivers by providing strategies to manage high costs, such as communicating with other family members and acknowledging cultural norms around caregiving financial decisions.

### Improving Future Intervention Delivery

Despite enthusiasm for the program, lower-than-expected attendance and completion rates suggest that caregivers may experience time barriers to participation [33]. Previous research suggests that live-in caregivers with intense caregiving duties lack time for web-based interventions [31]. Shorter or fewer sessions may increase participation and commitment. Further, paralleling other web-based studies, technical difficulties, audio issues, and schedule clashes impacted user experience and potential program adoption [33]. Caregivers also encountered logistical challenges that hampered their ability to complete assignments. Helping caregivers identify strategies to complete homework could foster greater program acceptance and treatment adherence. Examining participants’ expectations before delivering the program may benefit future implementations of CONFIDENCE [41].

### Limitations

Despite high acceptance levels among respondents, only 39% of caregivers who completed the CONFIDENCE intervention answered satisfaction survey questions. Moreover, the investigators did not record data on the proportion of participants who accepted qualitative interview invitations. A high nonresponse rate and lack of information about the qualitative response rate hinder a comprehensive assessment of caregiver experiences, as it is unknown whether survey completers had similar experiences to noncompleters. Future studies should prioritize strategies to enhance survey completion rates for a more representative analysis of the intervention’s impact, including providing a payment incentive for satisfaction surveys. The demographic composition of our sample also presents limitations. Most participants were women, such that male caregiver perspectives may not be adequately represented.

Additionally, most sessions were delivered in English, and most caregivers chose to complete interviews in English. A deeper analysis of language preferences and cultural differences could provide valuable insights. Coding in Spanish could uncover subtleties that go unnoticed, thus capturing nuances lost in translation and enriching the depth of our findings. Finally, while web-based delivery offers the benefit of reaching caregivers across geographic regions, it imposes a constraint by requiring a certain level of technological proficiency, restricting who can participate. Our study may not capture the acceptability experiences of caregivers without internet access, whose perspectives on financial circumstances may differ.

Finally, variation in session attendance and the voluntary nature of participation in surveys and interviews may have introduced self-selection bias. Participants who completed the evaluation components may have been more motivated or held more favorable views of the intervention. Missing demographic data in some survey responses further limits the ability to fully characterize the participant population. These factors should be considered when evaluating the generalizability and transferability of findings to the broader Latino caregiver population.

### Conclusions

Findings from this study suggest that the CONFIDENCE intervention is acceptable to Latino caregivers of persons living with dementia who seek to enhance their financial well-being and reduce out-of-pocket caregiving expenses. They also identified areas of improvement to make the program more accessible and more effective. This study contributes to the small but growing body of research on financial interventions designed to support caregivers, particularly within the Latino community. These caregivers have a higher risk of poor financial health while providing care, especially considering the rising rates of dementia among the Latino community [42]. Using culturally relevant psychoeducational interventions can enhance financial resilience and address the disproportionate costs borne by Latino caregivers.

## Supplementary material

10.2196/70471 Multimedia Appendix 1 The 7 component constructs of Sekhon’s theoretical framework of acceptability.

10.2196/70471 Multimedia Appendix 2 Interview guide.

## Abbreviations

CONFIDENCE: Confidently Navigating Financial Decisions and Enhancing Financial Well-Being in Dementia Caregiving
IRB: institutional review board
TFA: theoretical framework of acceptability
