# Supplementary material for: Acceptability of a Web-Based Financial Education Intervention for Latino Caregivers: Mixed Methods Evaluation
Source: JMIR Form Res. 2025 Jul 15;9:e70471. doi: 10.2196/70471 (PMC12282646; doi:10.2196/70471)
Supplement: Multimedia Appendix 1 [file formative-v9-e70471-s001.docx]

1. Affective Attitude: This construct refers to the emotional response or subjective feelings associated with accepting a particular intervention or behavior. Affective attitude reflects the individual’s positive or negative emotional response towards the intervention. For example, if someone perceives a recommended treatment as unpleasant or uncomfortable, their attitude may be negative, affecting their acceptance.
2. Burden: The burden construct considers the perceived effort, time, or resource investment required to adopt or engage with the intervention. It reflects how much individuals perceive the intervention as burdensome or demanding. The higher the perceived burden, the less likely individuals are to accept the intervention. Factors such as financial costs, time commitment, or lifestyle changes can influence the perception of burden.
3. Ethicality: Ethicality refers to the perception of the intervention’s moral or ethical appropriateness. It assesses whether individuals perceive the intervention as aligned with their moral values or ethical principles. The construct of ethicality acknowledges that people’s acceptance of an intervention may be influenced by their moral judgments. If individuals perceive the intervention as ethically sound, they are more likely to accept it.
4. Intervention Coherence: This construct relates to how individuals understand and find the intervention coherent, logical, or comprehensible. It reflects how well individuals can grasp the intervention’s purpose, mechanisms, or rationale. Lack of understanding or confusion about the intervention’s underlying principles may hinder acceptance.
5. Opportunity Costs: Opportunity costs refer to the potential sacrifices or foregone alternatives associated with a person accepting the intervention. It involves considering the trade-offs individuals who participate may have to make, such as giving up other activities, resources, or opportunities. If the perceived opportunity costs of accepting an intervention are high, individuals may be less willing to accept it.
6. Perceived Effectiveness: This construct evaluates an individual’s beliefs about the expected effectiveness or efficacy of the intervention. It refers to how individuals perceive the intervention as capable of achieving the desired outcomes. If people perceive the intervention as highly effective, they are more likely to accept it. On the other hand, if participants doubt its effectiveness, acceptance may be lower.
7. Self-efficacy: Self-efficacy reflects an individual’s belief in their ability to engage in the intervention successfully. It assesses a person’s confidence in their skills, knowledge, or competence to carry out the required tasks or behaviors associated with the intervention. Higher self-efficacy leads to greater acceptance, as individuals believe they can overcome challenges and achieve the desired outcomes.
