# Supplementary material for: Acceptability of a Web-Based Financial Education Intervention for Latino Caregivers: Mixed Methods Evaluation
Source: JMIR Form Res. 2025 Jul 15;9:e70471. doi: 10.2196/70471 (PMC12282646; doi:10.2196/70471)
Supplement: Multimedia Appendix 2 [file formative-v9-e70471-s002.docx]

- What do you think was the purpose of the CONFIDENCE program?
- What kinds of skills did you learn from participating in the CONFIDENCE program, if any?
- How did participation in CONFIDENCE affect your ability to seek out help about financial challenges you may be facing?
- How did participation in CONFIDENCE affect your ability to problem-solve financial challenges you may be facing?
- How did your participation in CONFIDENCE affect your ability to cope with stress from the high costs of caregiving?
- Do you believe that you are more confident about how to protect your financial wellbeing as a caregiver? If so, how has this affected your mental health?
- How has your approach to managing your financial wellbeing changed since attending CONFIDENCE?
- Please tell us your opinion if the CONFIDENCE program is or is not relevant to the experiences and needs of Latino family caregivers.
- Which aspects of the CONFIDENCE program did you find most beneficial to you as a caregiver and why?
- Which aspects of the CONFIDENCE program would you recommend changing and why?
- Is there anything else you would like to share about the CONFIDENCE program that I have yet to ask you?
